# “I’m not listening to my teacher, I’m listening to my computer”: online learning, disengagement, and the impact of COVID-19 on French university students

**DOI:** 10.1007/s10734-022-00854-4

**Published:** 2022-04-27

**Authors:** Charlotte Branchu, Erwin Flaureau

**Affiliations:** 1 University of Liverpool, Liverpool, UK; 2 EHESS-CMH, Paris, France

**Keywords:** Remote learning, Disengagement, French university, Weak institutions

## Abstract

In a French context where universities are thought of and analysed as “weak institutions” that leave learners to themselves, the effects of the pandemic present great risks to students who already face structural obstacles to their education. This qualitative paper is based on the experience of 19 students in a typical non-selective university in France, who have disengaged or have thought of dropping out during the pandemic. We examine how lockdowns and distance learning have impacted French students’ learning and living conditions unequally, unpacking ideas of student engagement and disengagement through a sociological lens looks at students within their social context and places importance on the role of institutions in “holding” students. By unpacking our participants’ narratives, we address the implications of online learning for educational justice and the long-term opportunities of students. Our analysis shows that students’ well-being and learning are entangled with an attachment to the institution, with seeing the worth, purpose, and recognition for what they do and the importance of emplaced learning to do so. We therefore highlight the importance of recognition and regulation in the learning process itself. Our findings allow us to also offer nuance to existing research and discourse about the French university system being a “weak” institution that forces individuals to be autonomous. The article also points to the strategies employed by students and teachers with a view to maintain engagement that contribute to considering solutions to help student retention for the sector as a whole and in our pedagogical practice.

## Introduction

No classes, no jobs, no social life: are young people greatly sacrificed to the health crisis? In France, one in six has stopped their studies, 30% have given up on accessing healthcare, and more than half are worried about their mental health. (France24).^1^

The well-being and prospects of students and young people have been a cause for concern globally during the COVID-19 pandemic, and France was no exception. Three national lockdowns were implemented: initially between 17 March–11 May 2020, then 30 October–15 December 2020, and finally 3 April–3 May 2021. Universities were completely closed during the first and second lockdown, but regulations were “relaxed” during the third lockdown in response to growing concerns regarding student mental health. France faced what was then described as a “suicide epidemic”: one case after another, student “distress” became a regular news item, accompanied with virulent critiques of government inaction (Le Monde, 2021a; Faridi, 2021). Increased attention was paid to the “fatigue” and suffering of both students and staff and their difficulty to adapt to online or hybrid learning (Le Monde, 2021b). These challenges were compounded by the fact that universities made little to no use of online learning environments prior to the pandemic. Discontent with the situation was noticeable on social media with popular hashtags used to voice political outrage during the first lockdown with #HonteUnivFrance (#ShameFranceUni) and student disengagement during the second with #EtudiantsFantomes (#GhostStudents). There is a general agreement that the lockdowns have accentuated existing inequalities, with more and more young people driven into poverty.

In the past year, numerous studies have scrutinised the impact of COVID-19 on French students’ experience of the pandemic. Whilst journalists have used first-hand accounts of a handful of students for storytelling,^2^ most emerging academic and policy research of sociological nature provides quantitative accounts, particularly of students’ social and material unequal conditions of living and learning^3^ during the pandemic. Difficult living conditions, technological/digital inequalities, and challenging family circumstances, it is clear that the students who have the worst end of the bargain are students from low socio-economic backgrounds and, more so, international students with an ethnic minority background who experience many intersectional oppressions (OVE, 2020a; Gosselin, 2022), in France as in other national contexts (Soria & Horgos, 2020; Aristovnik et al., 2020). COVID-19 as “the great social equaliser” is affecting students unevenly, with disadvantaged and marginalised students’ material security and mental health being disproportionally impacted (Aristinovnik et al., 2021; Chirikov et al., 2020; Husky et al., 2020).

We contribute to this emerging literature by providing an explanatory framework of how COVID-19 and the transition to online learning have contributed to educational exclusion and challenged educational justice. The article is empirically based on the experience of 19 students of different disciplines, year groups, and backgrounds, as well as teachers, in higher education. We examine how lockdowns and distance learning have impacted French students’ learning and living conditions unequally. When unpacking ideas of student engagement and disengagement, we take a sociological lens that does not exclusively look at students themselves but that also considers their social context and the role of institutions in “holding” students in. We contend that the challenges presented by online learning highlight the fact that recognition and discipline through co-presence are essential aspects of situating oneself in a learning environment and of learning itself. The lack of co-presence caused by the move to remote learning in turn affects students who are already vulnerable, in education in general and in higher education particularly. Through the lens of the pandemic, we offer nuance to existing research and discourse about the French university system being a “weak” institution. Our findings contribute to building solutions to ensure continuity of social justice through the transition to online learning and long-term opportunities of students who are already at a disadvantage within HE institutions and less likely to maintain engagement.

We deploy our arguments in three parts. Firstly, we look at the modalities of university attendance that students report—how they understand they got there, which matters for the meaning they attach to participation. Secondly, we analyse the challenges faced by students as a result of the move to online learning. We see that they report a disengagement and disentanglement from the institution, particularly linked to a loss of recognition, belonging, and meaning. Thirdly, we see how students have sought to “hold on” and explore the tactics put in place by teachers and the institution that have managed to maintain student engagement.

## The French context: universities as “weak institutions”

The French higher education (HE) system presents a few distinctive characteristics that set it apart from Anglo-Saxon models. Firstly, it can be considered “dual”. On the one hand are selective HE programmes that are both public and private institutions. Public-funded paths include IUT and STS,^4^ which are professionalising and usually target less privileged students, and CPGE^5^ and *grandes écoles*, attended by and made for “elite” students (see Darmon, 2015). Private institutions are mainly business schools and vocational schools (i.e. cooking schools). On the other hand are universities, which are non-selective public institutions and open to anyone with a high school diploma (Baccalauréat^6^). Though the sector is moving toward increasing, institutional selectivity through the ParcourSup system and the 2018 ORE law, selection often occurs *within* the university (Bodin & Orange, 2019). The university sector offers little vertical stratification between institutions and curricula. Stratification is highest between subjects and diploma level and exists mostly between Parisian and “provincial” universities, with non-formalised geographical and symbolic stratification among them. Attendance at university comes at a small fee, nationally fixed at €170 in 2021 for a year of a Bachelor’s degree/”License^7^”. The state offers bursaries to students based on need and provides all young people with an “aide au logement”, or housing aid, which reduces their rent. Living costs are not covered unless students are on a bursary. Students can benefit from access to cheap cultural spaces at university (vouchers, etc.) and affordable food options through the “RU” (university restaurant). Despite universities being more accessible than selective HE, these institutions remain less attended by the working and rural classes (OVE, 2020b). This “open system” is also changing, as the Édouard Philippe government introduced in 2018 the platform “ParcourSup”, which allows universities to exert greater power to select their students. Whilst we cannot go into the details of this reform here, we expect that it will have drastic consequences on the French HE system.

According to Bodin and Orange (2018:134), French universities “differ from their selective counterparts [in] that they offer relatively little supervision of students, whereas selective programmes tend to have smaller class sizes and closer supervision”. Universities can therefore be intimidating for students due to, among other things: limited contact hours, no monitoring of participation, and assessment period only twice a year. Lacking the cultural capital to feel confident if left to their own device, students from low socio-economic background will thus seek to enter professionalising selective institutions (IUT, STS) or give up the idea of higher education altogether.

French universities sustain a high level of failure, dropout, or change of academic orientation, especially in the first few years in HE. Whilst 39–41% of students obtain their Bachelor in 3 or 4 years (Repères et références statistiques, 2021), success rates heavily depend on students’ social origin and the type of Bac passed (the two being linked), with half *Bac général* holders graduating, compared to 16% from *Bac technologique* and 6% from *Bac professionnel* (ibid). Students with few social and scholarly assets can be discouraged by the lack of structure and the lack of human and material resources needed for close pedagogical support (Lapeyronnie & Marie, 1993; Felouzis, 2001). Success at university is also limited by the extent of self-discipline it demands and the wish for students to enjoy autonomy away from home and “have fun”, facilitated by the flexibility of the institution (Le Galès & Oberti, 1994). Due to these factors, students are only thinly linked to the institution. Mass education in France fails to develop a “student culture” observed by Becker among medical students that would allow them to negotiate workload (Becker & Geer, 1958), which is however observed in classes prépa (Darmon, 2015) or grandes écoles (Bourdieu, 1996). In an atomised milieu, students rely on themselves to reach out to teachers, manage their work, and make sense of class situations (Le Bart & Merle, 1997; Paivandi, 2011). For Sandrine Garcia (2010), the laissez-faire of universities actively reinforces inequalities of cultural capital and prior schooling capital. It deschools students and weakens their chances of success by letting individualities guide students’ experiences of education and long-term outcomes. Studies of French HE, as well as common representations, have clearly identified and critiqued universities as a “weak institution” (Felouzis, 2001). For Felouzis (ibid:14), this means “a system which does not impose clear collective goals on its members”, and in which the learning strategies students should deploy are left undetermined. Contrasting a “strong” institution which envelops its members toward a shared goal, identity, and clear sets of practices, such as *grandes écoles*, “weak institutions” are characterised by imprecision, incertitude, and a weak integration of its members. Students are atomised and experience a “freedom” that is lived as neglect. In practice, the weak institution asks “everything and nothing” of its students (ibid) in how they spend their study and leisure time: students *could* be involved as much or as little as they wish, with no clear rules, paths, or incentives.

## Educational justice and approaches to student (dis)engagement

Given the French context highlighted above, the concern with dropout and disengagement is particularly high when investigating universities and particularly crucial given the challenges of the global pandemic. Disengagement has been used to describe a range of phenomena, including dropout and educational exclusion. Whilst disengagement bears great influence on educational and public policy, its definition is contested, with ever-changing boundaries and parameters used to define it (for a review of this debate in the French context, see Bernard, 2015). The concerns are not only with inequalities in completion and attainment gaps, but student learning as a whole, and therefore also a concern for education sciences and pedagogues. Disengagement is often framed as an individual issue or personal lack, particularly in research from psychology or education studies, but sometimes also as an institutional failure. In the context of our study and current context, we must particularly account for disengagement that is a lot more “silent” than dropping out. Progressive disinterest, loose connection to one’s education, and loss of meaning of work whilst remaining “on the inside” have been studied by Broccolichi (2000) as “cognitive disengagement”. This is a central form of disengagement that is less spectacular and measurable than dropouts or absenteeism. We find this concept useful, but as sociologists, we problematise this as a process that occurs in a particular social context and base our analysis of this phenomenon in the socio-material context in which they occur for individuals. There is a large existing body of literature on student engagement and the student “experience”.^8^ However, these are not always sociological (Pötschulat et al., 2021) and often implicitly address debates to do with the students’ epistemic position as clients, partners of learning, citizens, or other (Dusi & Huisman, 2021). Engagement and disengagement literature broadly agree on the importance of meaning, belonging, and intention in successful learning experiences. In understanding engagement, Dubet (2016) identifies three main motives for students to invest themselves in particular paths or subjects which gives meaning to why they should care about their education: utilitarian (to obtain a degree), disinterested investment (intrinsic love for a particular subject), and the experience of socialisation (particular city, institution, friends, social environment). Rather than focus on intentionalist motives, Tarabini (2019) offers a model of educational inclusion that is focused on social justice particularly around 4 areas: care, recognition, redistribution, and representation. This holistic approach accounts for affective, cultural, economic, and political inequalities, respectively. Similarly, in this article, we take a sociological approach that accounts for dispositions as well as the interactional and situational context of practices. As Felouzis (2001) argues, sociology of education is often caught between a sociology of students (type of study, modes of learning, etc.—students without the institution) and a sociology of the university (institution without students). We seek to account for the students and their encounter or interaction with the institution and vice versa. In light of our current context, the adaptation (or lack thereof) of both toward the other is of primordial importance to this research.

## Methods

In this research, we used qualitative research methods to understand the lived experience of students. All semi-structured interviews were conducted in French and lasted between one and two hours and a half. For consistency, both researchers followed an interview guide that looked at the students’ trajectory in a chronological way (from decision-making about university and secondary schooling to the second lockdown). The questions were based around practices and established the networks, material, economic, and social circumstances of the participants’ situations, conditions, and decisions. We sought to establish how integrated in the university and other networks the students were, asking them about their spaces of sociability, friendship and family networks, participation in extra-curricular activities, class chats, and virtual learning communities. Affects were also a key part of our analysis. As researchers of embodied social inequalities, the “fleshy” aspects of experience were also important to our inquiry. Our positionality was accounted for in how it informed and shaped the interview situation and conversations. Interviews were conducted online through Zoom, which proved to be an interesting way to discuss online learning and being in the participants’ “space” without intruding.

### Sample and analysis

The field site is a university in a rural region of France. In many ways, the university context of the study can be described as “average”. The university is average in size and reputation and in the range of subjects offered, including some professionalising degrees. It is located in a city that is fairly populated, with approximately 140,000 inhabitants. It is the home of many bars, cultural venues, sporting opportunities, and relatively well connected to other important French cities by rail and road. Four other FE institutions are also in this city. Whilst the campus is within a mid-sized city, some of the participants were also in a more remote location, attached to the university but in a small town away from the city, in order to conduct their professional degree (DUT). Our field site is therefore typical in many ways, resembling the one studied by Felouzis (2001).

To recruit our participants, we originally wanted to reach out to students that had dropped out of their education. By speaking to a few academics and administrators across the institution, we found to our surprise that the rates were not particularly higher than other years (with a few exceptions, for example in Law). We are thus aware that we did not get to speak to this particular population that is hard-to-reach for many reasons, in spite of having a few chances that were inconclusive. We adopted snowball sampling to speak to students and teaching staff, all suggestions based on the assumption that it would fit our research topic (detailed in Table 1). Reach and greater quantitative representation is therefore a limit of the study. A longer exposure to the field would have yielded more diversity of participants, particularly international students, students of colour, mature students, those with caring responsibilities, and students enrolled in a broader range of disciplines. Snowball sampling however was helpful in building trust and rapport with participants, as they were referred to us by other participants whom they trusted. This was particularly important for the students, who might have felt intimidated by the process.

**Table 1:** Research participants

Name	Age	Ethnicity	Father’s occupation	Mother’s occupation	Study subject	Year
Matthieu	26	White	Engineer	School teacher	Social Sciences	5th
Ahmed	25	Black Senegalese	Cook	Secondary teacher	Social Sciences	2nd
Victor	20	White	Primary school teacher	Secondary school teacher	Social Sciences	2nd
Nathan	25	White	Restaurant owner	Restaurant employee	Social Sciences	5th
Louane	22	White	Unemployed/disabled	Unemployed/disabled	Social work	1st
Claire	21	White	Low skilled public servant	School intendant	Social work	1st
Nicolas	21	White	Farmer	Farmer	Chemistry	3rd
Baptiste	22	White	Firefighter	Social worker	Chemistry	3rd
Nora	25	Algerian/Italian	Psychomotor therapist (retired)	School headmistress	Pharmacology	5th
Lucas	21	White	Firefighter	Secretary	Social Sciences	3rd
Myriam	19	Moroccan	Shop owner	Manual worker	Sciences	1st
Khalid	23	NC	NC	NC	Law	5th
Laura	21	White	Intermediary public profession	Specialised educator	Social Sciences	3rd
Christian	20	Black	Unskilled manual worker	Unemployed	Sciences	2nd
Julien	22	White	Intermediary public profession	Secondary school teacher	Social Sciences	3rd
Célia	20	White	Artisan	Nurse	Social Sciences	2nd
Johanna	29	White	Skilled manual worker (deceased)	Unemployed	Social Sciences	2nd
Yasmine	20	White	Intermediary public profession	Unemployed	Social Sciences	2nd
Manon	20	White	HGV driver	Secretary	Social Sciences	2nd

We understand our participants’ social position simplistically on the table (parents’ profession) but accounted for variations and nuance in the interviews such as geography, racialised identities (including whiteness), schooling, siblings, grandparents, “cultural” aspects of class (sports, interests, relation to knowledge, and schooling), people’s understanding of their own social position, economic situation, and trajectories (movement within the social space). Nuances are tough to convey in such a short article and given our focus; therefore, we appeal to the sociological classifications used by statistical analysts, including INSEE and the Observatory for Social Inequalities, albeit with the objectivist shortcomings it presents

The participants we talked to were majoritarily from lower-middle classes or working classes, which is lower than the national average of participation in higher education. The distribution for university is of 9.1% rural classes, artisans, shopkeepers, and small business owners; 33.2% mid and high professional and intellectual occupations; 15.1% intermediary professions; 17.3% employees; 10.9% working classes; and 14.5% retired and unemployed (Observatoire des inégalités, 2020). Therefore, the findings of our study are much less representative of the middle and upper classes.

For the study, we also interviewed 7 members of teaching and administrative staff, including heads of department, programme directors, and welfare officers. Members of the pedagogical team we interviewed came from different departments, including some of the students. The results from these interviews cannot be discussed in the scope of this article but gave us elements of context for the students’ situations.

Interviews were conducted between January and April 2021 and subsequently transcribed. Data was analysed using a coding scheme that was continuously revised as we examined transcripts.

This study was approved by the Durham University’s ethics board. We guaranteed the anonymity, confidentiality, and welfare of our participants throughout the research process. Ethics were particularly important given the sensitive nature of the topic, particularly as the pandemic was still ongoing at the time. Both researchers being experienced in qualitative methods, we communicated tactfully and supportively before, during, and after the interview, ensuring that participants knew they could refuse to answer any questions and avoiding overly sensitive questioning. A restitution of the research to the participants, particularly teaching teams, was also planned.

## Results and analysis

### Going to university: uncertainty, respectability, and professionalisation

In this section, we analyse why and how our participants came to university. We particularly examine the type of engagement and expectations they had of universities as institutions, their relation to knowledge and school, their relation to social constraints and authority, the job market, and their life chances. This is revealing of their own social position and apprehension of learning and the institution. We find three dominant ways that determine our participants’ expectations and approach to learning and to their social identity as students; the dominant trajectories found in our participants are to have come to university: (a) “by default”; (b) to obtain social recognition, respect, or status; and (c) to professionalise and/or enter the job market. They are not mutually exclusive nor are they applicable to each participant at all times, as students can move between them; however, they are three primary ways in which participants express their interpretation of the reasons why they enrolled at university and become relevant when analysing their disengagement, as they become threatened. We will consider them in turn.

#### “By default”

Attending university was not every participant’s first choice. Baptiste (22, third year chemistry student), whose father is a firefighter and mother a social worker, wanted to continue his studies at a selective institution because he felt he needed educational “constraints”. By this, he and we mean structured learning akin to French secondary school, including discipline, clear timetables, monitoring, regular assessment, etc. Baptiste was told in high school that at university “nobody holds your hand, you have to be autonomous and if you can’t work on your own nobody will help you”, so he wished to apply for more rigid FE institutions such as classes prépa or IUT. However, he did not have the grades, so “[he] chose the easy path”. He eventually settled for the university nearest to his hometown, describing having ended up at university “by chance”. The discourse that one is left to their own device at university is predominant in secondary schooling, making it an “inadequate” choice for students who are less confident in their abilities to succeed in the absence of constraints, and those that have not incorporated the cultural capital that predisposes them to navigate the system, the social capital to develop these imaginaries (usually first generation scholars), or the confidence for autonomy (Reay, 2018). Students who internalised this discourse are often from low socio-economic backgrounds (Reay & Ball, 1998) and can struggle within an institution that offers weak constraints, what Sandrine Garcia describes as a “deschooling” institutional failing (2010). Similarly, Célia (20, second year social science BSc, mother nurse, father employed artisan) says that she is “not meant to be here”. Loving fashion, she wanted to do a BTS but was told by her parents and teachers that “[she] wouldn’t have the level [grades]” and that she’d have a better chance at university. Understanding the university as “for slackers”, she did not attend many classes and did not understand how to “do university” (Coulon, 1997). She enrolled into her social science degree “out of spite” after hesitating with economics, but in her first year: “I discovered a true passion for [social science]”, she says with surprise.

Whilst some students come in with a pre-defined “path” in mind, research has shown that many do not, discovering career prospects and the value of their degree as they go, as in the case of both Célia and Baptiste, who also got interested in his studies at the end of his second year with the help of teachers and guided by his grades (Felouzis, 2001). However, these students do have the understanding that tertiary education is something worthwhile to pursue and are encouraged to do so. Baptiste’s parents: “strongly encouraged me to do further studies, especially my dad, though he didn’t do any, I guess that’s why he pushed me to do some. Then they don’t care because they really don’t get what I do [laughs] […] But yeah they were very happy that I went on to further education and encouraged me to do so”. These individuals often present what Bourdieu calls “cultural goodwill”, in upholding the social and symbolic value of a degree. Students in this category see the value of hard work but are not attached to a particular path or do not always believe in the intrinsic value of the topic they study.

#### Social recognition, legitimacy, respect, and status

Gaining social recognition is an important feature of obtaining a degree for our interviewees. This can be in the form of earning respectability and social status. Johanna (29, second year social science, married to a HGV driver, 2 young children, stay-at-home mother, father skilled manual worker [deceased]) dropped out of school before finishing secondary education and obtained her Baccalauréat equivalence later in life. She expressed feeling devalued because of it: “it wasn’t really me feeling this way, it was society making me feel this way. That since I hadn’t done any studies, I didn’t matter, my opinion didn’t matter, nothing that I could say or do. So that really made me want to pass my Bac equivalence and study”. Ahmed expresses a similar wish to be *seen* as someone who should be respected. After dropping out of his first Law degree in his third year and working as a seasonal worker, he goes back to his studies, as he puts it, “to earn respect” (25, second year social science, black Senegalese, father sous-chef in Geneva, mother high school teacher of economics in Senegal (divorced), 2 siblings). In Bourdieusian terms, obtaining a degree is a way to convert cultural capital into symbolic capital (1986), particularly for some of our participants who were already experiencing themselves as socially “disqualified”—Johanna as a working-class mother and Ahmed as a racialised man with an immigration background. Education here is seen as a way to (re)gain respect and respectability or even status and to achieve social mobility.

#### Professionalising and entering the job market

Few participants came to their degrees with a particular professional objective. This is the case of social work students, who switched to this degree after originally enrolling in a different one,^9^ and students in social science who “just need any degree” to enter teacher training. Manon, who is from a stable working-class family, came to university knowing she wanted to be a schoolteacher and needs an undergraduate degree to access her teacher training masters. Coming from a scientific Bac, she hesitated to pick her subject but settled on social sciences, thinking she had enough maths and physics. She has performed well as she has discovered that she likes the subject, although she retained her professional goal. Students like Manon follow what Dubet qualifies as a “utilitarian” motive to stay engaged (2016): she has the clear objective of obtaining a degree, and she simply wishes to not be bored and get good grades. As described by Fernex et al. (2015:418), “the [French] university system operates on a strict diploma-securing logic (scoring the average [to pass])[…] [Some students] seem to calculate their investment in time with a view to effectiveness, i.e. obtaining the degree which is a relatively unified and non-differentiated product”. In our study, we saw that student adopting this approach—whether the degree is professionalising or not—were more flexible and thus able to adapt more easily to the challenges of studying at university as well as those presented by the move to online learning that we discuss below.

The reasons students identified as why they came to university and “engaged” in the first place are crucial to understand what the switch to online learning and lockdown threatened and therefore which students were more affected by those measures and why.

### The lived experience of lockdown: challenges, disengagement, and loss of meaning

Our participants had been at university for a year or longer when the first lockdown started and were accustomed to independent learning. However, participants’ experience of online learning during lockdown turned out to be very different from what they were used to, particularly in the drastic decrease in the structures and social regulations that usually help them to regulate their work. This anomic situation weighed gradually on their motivation to carry out their university work and, above all, to find meaning in their “student job” (Coulon, 1997,10). Victor shows this loss of meaning in recounting that:

At the beginning I was trying to follow, but then there was a long phase where I dropped out completely. To me, there was no use in what we were doing. [...] Since we no longer have a structure, we no longer have any obligation, we are no longer monitored [for attendance], I just picked and chose what suited me the most. And yet, I was freaking out, I thought it would be annoying if I didn’t [pass] my year. (Victor)

As Victor points out, the absence of social regulation, such as framework or obligation, determines the lived experience of disengaging from distance learning. As such, the classroom constitutes a constraining framework that usually regulates behaviour. In a remote learning situation, the ability to get to work and maintain studious attention depends mainly on the student, who must be able to show more self-restraint. Nevertheless, students report difficulties in staying focused within their domestic space^11^ where they are disturbed by a range of factors. Célia highlights what many say, that “there were lots of disruptive elements around me: my phone, the TV, my boyfriend [laughs]. It was too complicated. It’s complicated enough to stay focused for two hours on the same subject, so here at home, it’s hell”.

The fact that the organisation of the workspace is often tough to negotiate, particularly for low-income students, is corroborated by quantitative studies that look at learning conditions and economic hardship (i.e. Soria & Horgos, 2020). Our participants often did not have a space of their own to study, or when they lived alone, a study space (desk, table). The encroachment of “work” on “life” was highlighted as an issue, not only for themselves but also the people around them, mostly due to classes going on until 8 pm, for example. Having to eat in front of the live class (cameras off), with no breaks between classes to move or stretch was an issue. This was made worse by the disappearance of leisure spaces and other spaces of socialisation, and “personal” or intimate space was reportedly challenged if not attacked. For some, the loss of in-person teaching and a regular rhythm also meant a loss of time, difficulties in sleeping, and seeing time pass, weeks “blurring into one another” (Remi).

This “ritualistic presence” is usually limited to lectures (Bourgin, 2003) that student attends without having to pay too much attention. In lockdown this is extended to teaching in small groups such as tutorials. As Victor points out, online you don’t have the constraint of “you’re in class, you have to follow”. In a seminar, “usually the teacher is in front of you, he tells you to work”; even if one does not want to, they would get recalled to the shared socio-material space.

Remote classes give us a lot of freedom, contrary to face-to-face because in presence teachers… you’re in a room and you feel surveilled so you feel like you have to work whereas now we’re at home, nobody sees what we do. The hardest thing is to listen to your computer [...] I’m listening to the teacher in my opinion, I’m listening to my computer. There isn’t this physical contact.

This dichotomisation of the computer/digital versus humans was common to most participants, both students and teachers. These remarks emphasise the *emplaced* (Pink, 2011) nature of learning, where it is not only in a particular space, but also requires a particular physical co-presence that captures or directs attention. Many students indeed identify a major struggle of online classes is not knowing how to *select* information, something usually done through tone and body language in class. When asked to describe a “good class”. students point to when a teacher manages to put some “humanity” back in, for example when the teacher is very humorous or simply has a cup of coffee, making them “human”. For students as well as for teachers, online learning will (unfortunately for all) constrain pedagogical action toward a unilateral monologue which promotes iniquitous modes of learning and symbolic violence (Bourdieu et al., 1965).

If students live this absence of interaction with teachers as an absence of constraints that challenges their ability to get to work, it also represents for some a loss of valorisation. Here, students are deprived of the symbolic gratifications they used to obtain when they attended class or submitted homework. Some interviewees expressed the feeling of “no longer mattering to teachers” (Ahmed). In other words, their experience of lockdown is accompanied by a “denial of recognition”: the absence or the strong decrease of social interactions with teachers can no longer engage the student through “providing them with proof of their existence and their valuation through the gaze of the other or others” (Paugam, 2018 (2008): 64). For many students, this leads to a loss of motivation and meaning:

I had the impression to work for nothing. […] I thought ‘until when do I have to do this?’. So I completely lost my motivation (Johanna)

Participants show that an even weaker institutional presence of university time during lockdown increased the “anomic effects of deschooling” (Garcia, 2010, p. 54) that supposedly occur at non-selective universities. The imbalance between free time and university time, already ordinarily tending toward a “dilution” (Millet, 2003, p. 163), was accentuated, and the difficulty of resisting deregulation, disengagement, or dropping out was heightened. If in a normal learning context student “adopt the lowest work norm that the institution allows” (Monfort, 2000: 74), the margin of autonomy that teachers permitted during lockdown further reduced these work demands.

Lockdowns have shed light on the regulating elements that usually operate at university that new students, used to the much stricter structure of secondary schooling, do not usually notice. The absence of classes, collective time and rituals such as exams, and the socio-material, embodied dimensions of learning all participate in building a sense of belonging and in meaning-making—both of which are essential to student retention in higher education. Although the French university is indeed a “weak institution” with a laissez-faire approach, students’ experience of lockdown learning is revealing of the mechanisms of social regulation that implicitly occur. Indeed, if distance learning is a challenge for students, it is because it calls into question a range of norms, rules, and behaviours that were previously tacit and silently contributed to the socialisation of students and their continued engagement in higher education, all of which already only tenuously keeping some students at university. The challenges highlighted above increase existing challenges faced by students of lower socio-economic backgrounds and marginalised students, particularly those who came to university “by default” or seeking greater social recognition. The purpose and meaning of their presence are called into question by the absence of legitimising recognition and any robust, interactional frame. In this group are students that possess less social, scholarly, and material capital to face these obstacles.

### Coping with lockdown: students, teachers, and institutions in the face of online learning

Whilst all student participants describe having “let go” and disengaged, students collectively and individually came up with tactics and strategies to cope with the challenges of the pandemic.

To hold on, some students sought to establish external constraints for themselves, particularly male participants. This is a result of a desynchronisation with the social time of the group and of regular living hours. Male participants struggled to keep a “regular rhythm”, struggling to go to sleep and wake up early, losing track of time, and mostly using screens to pass the time without being able to get to work. Faced with this, these young men tried to impose on themselves a more constraining framework, mostly through changing their living conditions and living with others (parents, partner, friends). Victor spent the second lockdown with one of his friends: “that way, I thought it was going to motivate me. We’d get up, have our classes, we’d hold on a little bit”. However, as Victor says: “at the beginning it was fine, but then it worked less and then not at all.” Indeed, the external constraints that work are instead those imposed by the institution: upcoming exams, emails from teachers, in-class incentives. This can be as mundane as having a technological advantage: “[In online classes] it was just the two of us with another guy that had their mic on and interacted with the teacher […] and since it was only two of us that could speak, we had to pay attention” (Nathan).

Faced with the progressive decrease of learning regulations during lockdown, female students differed from their male peers in the self-discipline they exerted. For instance, they paid great attention to their working environment, most female participants reporting that they had changed the layout of their flats to motivate themselves, even if at a small scale. They would also put in place a timetable, to respect deadlines but also to structure their day: “as soon as we have the assessments, I make a calendar where I indicate this day I do this, that day I do that. Basically, I would revise for one exam a day” (Manon). This self-discipline among female students suggests, as observed elsewhere, a greater attention among women and girls to satisfy institutional expectations throughout their schooling career (Djider & Robin, 2003), often rooted in family expectations (Baudelot & Establet, 1992). Despite this, or perhaps because of it, female students express a progressive fatigue after repeated lockdowns, principally linked to the emotional labour of staying afloat and, mostly, because of the inability to anticipate the future and how long the situation will last.

In the successive lockdowns, cohort Facebook and Whatsapp groups (that did not exist before^12^) allowed students to share notes but also discuss their experiences. Confronted with the issue of understanding content and getting assessments done, students collectively deploy modes of learning that define what is worth learning, handing in, and what is not. This was particularly the case for our participants in chemistry, where there is a “supportive culture” (Nicolas), reportedly with no competition, where the group works toward the common goal of succeeding in their degree and proceeding to master’s level together. In these online spaces, “if anyone asks for help, they will get it”. For students in these networks, continuous exchanges with other students are a means to maintain a minimum of engagement and to have perspective on shared challenges: “we’d realise that nobody had worked and that we shouldn’t work until 3am to hand something in. It was comforting. That I wasn’t the only one and everyone was in the same situation” (Victor). The difficulties of learning during lockdown that all our participant students encountered contributed to the emergence of a “student culture” (Becker & Geer, 1958) that allowed them to negotiate the work and workload. Through social networks, student groups form an integrated milieu where many face common issues and interact in order to find solutions, where people who face similar issues on a daily basis can handle them as a group (Becker & Geer, 1958).

The collective conception of learning expectations put in place by the students themselves is also used in the negotiations that student representatives have with staff:

Since during the first lockdown we reproached teachers not to have given us enough work and not having graded us properly, during the second lockdown they bombarded us with work […] my eyes were done, my back was aching. We spent our lives in front of the screen [...] At some point, I sent an email to the Undergrad studies coordinator, we talked, and they tried to improve things. It helped, even if we still had loads of work (Johanna)

Student representatives could feed back the collective feelings of their peers, notably the challenge to keep up. The emergence of a student culture that was possible because of lockdown gave more legitimacy to the demands of student groups that also found sympathy among teachers. This collective strategy would not “normally” happen, in an institution where students are atomised and rely on a small group of close friends as support networks. Research by Raaper et al. (2021) has also highlighted the prevalence of close networks for non-traditional university students in finding support and help, as opposed to professional services or weaker ties.

Indeed, for students who are more isolated, this collective adjustment to university work is harder. It is particularly the case for master students who entered university as another lockdown hit and before they could socialise extensively with the group, notably Ahmed. Unfamiliar with his peers, his understanding of work expectations and work norms rely mostly on teachers’ demands. He could not keep up with the pace, “physically, I was exhausted, literally”. He progressively gave up and fell into a depression: “it was insurmountable, and I decided to stop completely and start again in January”. It was only in a Twitter thread on student ill-being that he realised that “I’m not on my own. There wasn’t anything deeply wrong with me. That it [struggling] was normal”.

As well as external constraints, self-discipline, and spaces of solidarity and belonging, an important factor that helped students’ well-being and engagement during lockdown were demonstrations of “care”, particularly in interactions with staff.

For Baptiste, the second lockdown proved particularly difficult; he fell in a deep depression following four deaths around him, a break-up, family issues, his source of income disappearing (he had been a judo instructor), and the fear of struggling with learning online once more. He describes this time as having “failed everything”. He did not talk to anybody and was able to hide his issues to his friends, including the fact that he was forced to eat only once a day, sometimes at the soup kitchen because “[he] had to choose, either I’d [drive] to class or I’d eat, so obviously I chose to go to class”. Baptiste, a “by default” student, was deeply depressed and disengaged until a close friend and classmate noticed his behaviour and told their teacher something was going on. The teacher called him in to talk and “this is when things started changing”. She had done some research about what kind of psychological help he could access and gave him some phone numbers to call.

It relieved me, subconsciously I think, because even if I wasn’t the one to talk to her about it, that [my friend] told her about it without any detail, just to know that someone knew, it was a relief.

Baptiste held these phone numbers in his hand several times but never managed to make the call. However, his teacher’s gesture helped. “I would have totally given up if she hadn’t noticed something was wrong and hadn’t been there to give me a little push. So yeah, I don’t think I’d still be at uni if there hadn’t been that small detail”.

To be “pulled” back in requires an institutional effort but also an interpersonal exchange that signals to the individual that they “count for”/matter for someone (Paugam, 2008). We analyse care as an act of recognition that students notice and which helps (most of) them feel the hold of the institution—and hence holding on to it. This is also evident in the case of Ahmed who declares, upon receiving an email about his failure to submit coursework, that he “was touched that someone had noticed”. This particularly mattered for those students who are at university “by default” or are in search of recognition, as these acts of “care” gave them confidence and meaning to persevere in their education.

## Conclusions

In this article, we have explored how the pandemic and switch to online learning has affected students in French higher education. Whilst French universities can be understood as “weak” institutions, we argue that online learning has exposed the existence of more constraints, structures, and regulations than previously perceived by students, academics, parents, and circulated in common sense discourse. Existing studies about French universities tend to emphasise what it “lacks” (funds, regulations, cohesiveness, etc.), where students are abandoned by teachers who, by lack of resources or motivation, would neglect the pedagogical relation. Our research shows that there is indeed regulation and support at play. The disruption provoked by COVID-19 allows us to see the institutional hold that pre-existed in universities. An attention to everyday practice showed that the pandemic put in peril the relative institutional “strengths” of universities that were interactional, mundane, and spatialised.

As these constraints were stripped away, a particularly important aspect of learning and attending university was also eroded, that of care and social recognition. Our analysis has shown that students’ well-being and learning is entangled with an attachment to the institution, with seeing the worth, purpose, and recognition for what they do. This was particularly important since students who rely on recognition and validation to legitimise their place in higher education and to engage in the learning process are students who are already disadvantaged or have been socially disqualified—those there “by default” and in search of legitimacy.

We also note that students were proactive in trying to remain engaged. Individual strategies, which are often promoted and framed as “resilience”, failed almost in every case. Instead, social recognition, care, and the re-establishment of (some) institutional constraints have been effective in pulling students back or alleviating some of the challenges they faced. Although there are several ways in which students disengage or are re-attached to the institution, it is clear from our research that any solution is a *social* solution.

This theoretical conclusion thus offers a practical contribution for greater social justice in teaching by paying particular attention to issues of recognition and legitimacy for socially disadvantaged students. Such findings are transferable to pedagogical practice in other national contexts, seeking to maintain student engagement, not as neoliberal incentive, but to strive toward a human, relational, and pedagogically effective teaching praxis. In fact, this research has guided our practice as teachers in different national context in significant ways.

The university sector is not a singular entity, and, within our own case study, departments cared greatly in the response they could offer to students. We regret not being able to include the tremendous efforts deployed by the teachers we spoke to and the staff that supported them and the students.

However, during this research, we were astounded and humbled by our interviewees’ gratitude to have been listened to. Most expressed that the interview was cathartic or even healing and sometimes transformative—some reporting having found meaning and taste for their studies again. As Nora mentions at the end of her interview: “it’s incredible to have this format and be able to just talk with someone like this. I’ve been saying to people, it’s good to try and make some efforts on your own but at some point just talking to a human changes everything. I don’t know if you saw it that way but this [interview] is insane psychological support, just asking questions and letting people speak. Nobody does it. […] It just does a world of good to feel considered”. What we analysed here was a silent means of disengagement, one less quantifiable than drop-out rates but that constitutes a qualitative issue that is pressing to address. With this study, we hope to convey the methodological, political, and representational importance to listen.

## Footnotes

1 [our translation] France24 news story, 26/01/2021. Accessible: https://www.france24.com/fr/émissions/le-débat/20210126-covid-19-en-france-les-étudiants-en-détresse

2 For a prime example, see Kronlund (2021).

3 From the University of Burgundy (Accessible: https://iredu.u-bourgogne.fr/wp-content/uploads/2020/06/20020.pdf); Amiens (Accessible: http://amiens-sociologie.fr/index.php/enquete-sur-le-confinement-des-etudiants-de-lupjv/?fbclid=IwAR0TfXLyCtPhyknEdPNKh5A_JigYOVtpdNlVQTMvDlzNc3ZllKlS_KvONrM); the EHESS (Accessible: https://masterspps.hypotheses.org/445?fbclid=IwAR2w6TWhe8U3mMoYOWpHVEujAQVfDG7GJLUJswdVD-2OhIcOP-H-9_s5k2g); and from the Observatory of Student Living (Accessible: http://www.ove-national.education.fr/enquete/la-vie-detudiant-confine/); to mention the most notable ones.

4 Respectively: University Technological Institute; Superior Technician Section; and Superior Technician Certificate.

5 Classes Préparatoires aux Grandes Écoles, classes to prepare for entrance exams to grandes écoles. Thereon referred to as “classes prépa”.

6 Prior to entering university, secondary schooling includes tracking, particularly for the last 2 years of study. Students will most often complete either a Baccalauréat général (with 3 choices of scientific, literary, or social and economic sciences track) or professional. In the analyses, we will refer to the Baccalauréat occasionally as “Bac”.

7 This applies to home students. A reform voted in 2019, not without resistance, introduced international student fees. These fees vary according to institution, level of study, specialism and nationality, and/or residence status of the student, ranging from around 3,000 to 10,000 euros for a public university degree.

8 For a comprehensive, critical review of approaches to “engagement”, see Zepke (2017).

9 This speaks to Bodin and Orange’s (2018) understanding of university drop-out as regulation, in that it is in no way a personal failure but allows re-orientation and professionalisation.

10 Coulon, analysing the transition from school to university, describes how students need to learn some implicit rules and methods to succeed at university. He understands this adaptive learning process to correspond to the “work” or craft that students need to deploy. Coulon thus classifies this as a “student job” (*le métier d’étudiant*) or rather the job of being a student.

11 Determined by the students’ personal circumstances and material living and learning conditions.

12 Some were initiated by tutors and teachers.

## References

[CR1] Aristovnik, A., Keržič, D., Ravšelj, D., Tomaževič, N., & Umek, L. (2020). Impacts of the COVID-19 pandemic on life of higher education students: A global perspective. *Sustainability,**12*(20), 8438. 10.3390/su12208438.

[CR2] Baudelot, C., & Establet, R. (1992). *Allez les filles !* Seuil.

[CR3] Becker HS, Geer B (1958). Student culture in medical school. Harvard Educational Review.

[CR4] Bernard P (2015). Le décrochage scolaire.

[CR5] Bodin R, Orange S (2018). Access and retention in French higher education: Student drop-out as a form of regulation. British Journal of Sociology of Education.

[CR6] Bodin R, Orange S (2019). La gestion des risques scolaires. Avec Parcoursup, je ne serais peut- être pas là. Sociologie.

[CR7] Bourdieu P, Richardson J (1986). The forms of capital. Handbook of Theory and Research for the Sociology of Education.

[CR8] Bourdieu P (1996). State nobility.

[CR9] Bourdieu, P., Passeron J-C., & Saint Martin (de), M. (1965). *Rapport pédagogique et communication, Cahiers du Centre de sociologie européenne (2)*, Mouton.

[CR10] Bourgin, J. (2003). Les effets de la massification sur les pratiques d’enseignement universitaires, à travers une approche monographique de deux DEUG “réformés”, *Communication à la journée d’étude du RESUP*.

[CR11] Broccolichi, S. (2000). Désagrégation des liens pédagogiques et situations de ruptures. *VEI Enjeux, le décrochage scolaire**: **Une fatalité?*, *122*:36–47.

[CR12] Chirikov, I., Soria, K. M., Horgos, B., & Jones-White, D. (2020). *Undergraduate and graduate students’ mental health during the COVID-19 pandemic*.​ SERU Consortium, University of California – Berkeley and University of Minnesota. ​https://cshe.berkeley.edu/seru-covid-survey-reports

[CR13] Coulon, A. (1997). *Le métier d’étudiant : l’entrée dans la vie universitaire*. PUF.

[CR14] Darmon M (2015). Classes préparatoires: La fabrique d’une jeunesse dominante. La Découverte.

[CR15] Djider, Z., & Robin, I. (2003). “Motivation et performance scolaires : les filles creusent l’écart”, *INSEE Première*, 886.

[CR16] Dubet, F. (2016). École : la question du sens. In M. Fournier (ed.), *Éduquer et Former. Connaissances et débats en Éducation et Formation *(pp. 401–411) Éditions Sciences Humaines.

[CR17] Dusi D, Huisman J (2021). It’s more complex than it seems! Employing the concept of prosumption to grasp the heterogeneity and complexity of student roles in higher education. Higher Education.

[CR18] Faridi, R. (2021). Coronavirus: French students highlight pandemic’s mental health toll. *BBC News*. Available online: https://www.bbc.co.uk/news/world-europe-55716340 [Accessed 16/03/21].

[CR19] Felouzis G (2001). La condition étudiante.

[CR20] Fernex A, Lima L, de Vries E (2015). Exploring time allocation for academic activities by university students in France. Higher Education.

[CR21] Garcia S (2010). Déscolarisation universitaire et rationalités étudiantes. Actes De La Recherche En Sciences Sociales.

[CR22] Gosselin, A. (2022). Chapitre 7. Les inégalités intergénérationnelles à travers le prisme du statut migratoire: Conditions de vie des jeunes immigrés et descendants d’immigrés pendant le premier confinement. Dans : Yaëlle Amsellem-Mainguy éd., *Jeunesses**: D’une crise à l’autre *(pp. 149–164). Presses de Sciences Po.

[CR23] Husky MM, Kovess-Masfety V, Swendsen JD (2020). Stress and anxiety among university students in France during Covid-19 mandatory confinement. Comprehensive Psychiatry.

[CR24] Kronlund, S. (2021). Pandémie : des étudiants sacrifiés. *France Cultu*re. Available online : https://www.franceculture.fr/emissions/les-pieds-sur-terre/pandemie-des-etudiants-sacrifies

[CR25] Lapeyronnie, D., & Marie, J-L. (1992). *Campus blues : les étudiants face à leurs études*. Le Seuil.

[CR26] Le Bart C., & Merle, P. (1997). *La citoyenneté étudiante. Intégration, participation, mobilisation*. PUF.

[CR27] Le Galès P, Oberti M (1994). Lieux et pratiques sociales des étudiants dans la ville : Rennes, Besançon et Nanterre. Annales de la Recherche Urbaine.

[CR28] Le Monde. (2021a). Nous disposons de données qui questionnent l’émergence possible d’une épidémie de suicides chez les étudiants. *Le monde*. Available online: https://www.lemonde.fr/idees/article/2021a/02/04/nous-disposons-de-donnees-qui-questionnent-l-emergence-possible-d-une-epidemie-de-suicides-chez-les-etudiants_6068774_3232.html?fbclid=IwAR1Lp8xtwdGMsBHMgNdvYEIm_FHB2pNV3qs0KdFItN2nrHpXVwxfqA7ExIY [accessed 28/07/21].

[CR29] Le Monde. (2021b). Covid 19: des universités en souffrance. *Le Monde*. Available online : https://www.lemonde.fr/idees/article/2021b/01/09/covid-19-des-universites-en-souffrance_6065728_3232.html

[CR30] Millet, M. (2003). *Les étudiants et le travail universitaire*. PUL.

[CR31] Monfort V (2000). Normes de travail et réussite scolaire chez les étudiants en première année de sciences. Sociétés Contemporaines.

[CR32] Observatoire de la vie Étudiante (2020a) ‘Student life key facts’. Online Report. Accessed 11 March 2022. http://www.ove-national.education.fr/publication/student-life-key-facts/.

[CR33] Observatoire des inégalités. (2020b). Les milieux populaires largement sous-représentés dans l’enseignement supérieur. Online resource. Available: https://www.inegalites.fr/Les-milieux-populaires-largement-sous-representes-dans-l-enseignement-superieur

[CR34] Paivandi, S. (2011). La relation à l’apprendre à l’université, *Recherches Sociologiques et Anthropologiques,**42*(2). 10.4000/rsa.730

[CR35] Paugam, S. (2008). *Le lien social.* PUF.

[CR36] Pink S (2011). From embodiment to emplacement: Re-thinking competing bodies, senses and spatialities. Sport, Education and Society.

[CR37] Pötschulat M, Moran M, Jones P (2021). ‘The student experience’ and the remaking of contemporary studenthood: A critical intervention. The Sociological Review..

[CR38] Raaper, R., Brown, C., & Llewellyn, A. (2021). Student support as social network: Exploring non-traditional student experiences of academic and wellbeing support during the Covid-19 pandemic. Educational Review.

[CR39] Reay D (2018). Working class educational transitions to university: The limits of success. European Journal of Education..

[CR40] Reay D, Ball SJ (1998). ‘Making their minds up’: Family dynamics of school choice. British Educational Research Journal.

[CR41] « Repères et références statistiques 2021 », DEPP, September 2021.

[CR42] Soria, K. M., & Horgos, B. (2020). *Social class differences in students’ experiences during the Covid-19 pandemic*. Centre for Studies in Higher Education.

[CR43] Tarabini A (2019). The conditions for school success: Examining educational exclusion and dropping out.

[CR44] Zepke, N. (2017). *Student engagement in neo-liberal times*. Springer Nature.

